# Appraisals by Health Technology Assessment Agencies of Economic Evaluations Submitted as Part of Reimbursement Dossiers for Oncology Treatments: Evidence from Canada, the UK, and Australia

**DOI:** 10.3390/curroncol29100602

**Published:** 2022-10-13

**Authors:** Graeme Ball, Mitchell A. H. Levine, Lehana Thabane, Jean-Eric Tarride

**Affiliations:** 1 Department of Health Research Methods, Evidence, and Impact, Faculty of Health Sciences, McMaster University, Hamilton, ON L8S 4L8, Canada; 2 The Research Institute of St. Joe’s Hamilton, St. Joseph’s Healthcare Hamilton, Hamilton, ON L8N 4A6, Canada; 3 McMaster Chair in Health Technology Management, McMaster University, Hamilton, ON L8S 4L8, Canada

**Keywords:** oncology, health technology assessment, methods, CADTH, NICE, PBAC

## Abstract

Publicly funded healthcare systems, including those in Canada, the United Kingdom (UK), and Australia, often use health technology assessment (HTA) to inform drug reimbursement decision-making, based on dossiers submitted by manufacturers, and HTA agencies issue publicly available reports to support funding recommendations. However, the level of information reported by HTA agencies in these reports may vary. To provide insights on this issue, we describe and assess the reporting of economic methods in recent oncology HTA recommendations from the Canadian Agency for Drugs and Technologies in Health (CADTH), National Institute for Health and Care Excellence (NICE), and Pharmaceutical Benefits Advisory Committee (PBAC). Publicly available HTA recommendations and reports for oncology drugs issued by CADTH over a 2-year period, 2019–2020, were identified and compared with the corresponding HTA documents from NICE and the PBAC. Reporting of key model characteristics and attributes, survival analysis methods, methodological criticisms, and re-assessment of the economic results were characterized using descriptive statistics. Dichotomous differences in the methodological criticisms observed between the three agencies were assessed using Cochran’s Q tests and substantiated using pairwise McNemar tests. Chi-squared tests were used to assess the dichotomous differences in the reporting of methods and explore the potential relationships between categorical variables, where appropriate. HTAs published by CADTH, NICE, and the PBAC consistently reported a broad spectrum of descriptive information on the economic models submitted by manufacturers. While common economic evaluation attributes were well-reported across the three HTA agencies, significant differences in the reporting of survival analysis methods and methodological criticisms were observed. NICE consistently reported more comprehensive information, compared to either CADTH or PBAC. Despite these differences, broadly similar recommendation rates were observed between CADTH and NICE. The PBAC was found to be more restrictive. Based on our 2-year sample of oncology, the HTAs published by CADTH matched with the corresponding HTAs from NICE and PBAC; we observed important variations in the reporting of economic evidence, especially technical aspects, such as survival analysis, across the three agencies. In addition to guidelines for HTA submissions by manufacturers, the community of HTA agencies should also have common standards for reporting the results of their assessments, though the information and opinions reported may differ.

## 1. Introduction

Publicly funded healthcare systems in Canada, the United Kingdom (UK), and Australia use a health technology assessment (HTA) to inform drug reimbursement decision-making. In order for a new medication to be publicly reimbursed in these countries, pharmaceutical companies are required to submit a reimbursement dossier, which includes, at minimum, the clinical data used for regulatory approval, as well as a model-based economic evaluation to demonstrate the value for money of this new therapy in a given therapeutic area. Following a critical review of the manufacturers’ clinical and economic evidence by the HTA agencies, the HTA appraisals and funding recommendations associated with these products are publicly posted on the HTA agency websites.

It has been shown that drug funding recommendations by HTA agencies may differ, due to differences in political priorities [1], agency mandates [1], processes and procedures [2,3], or healthcare systems [2]. However, the level of reporting and appraisal by HTA agencies of economic models submitted by manufacturers for reimbursement appears to not have been previously investigated. This is important for a few reasons. First, while the economic guidelines that manufacturers are required to follow for drug submissions to each HTA agency are relatively detailed [4,5,6], there are no explicit guidelines that HTA bodies are required to follow in the reporting of their economic appraisals of manufacturers’ reimbursement submissions. Secondly, physicians, patients, or patient associations, as well as the general public, rely on the public information provided by these HTA agencies to understand the rationale behind the funding recommendations made by the HTA agencies. Finally, in Canada and, to a lesser extent, the UK, the appraisal of the economic evidence serves as a basis for price negotiations between the manufacturers and public plans. To fill a gap in the literature, we sought to answer the question of whether HTA agencies in Canada, the UK, and Australia are consistent in their reporting and appraisal of the economic evaluations submitted by drug manufacturers for the reimbursement of oncology medications. Building on our previous work regarding economic evaluations in oncology in the published literature [7], we hypothesized that consistency would be observed for oncology medications evaluated by the three HTA agencies, due to the same product being assessed, based on the same or similar clinical data.

## 2. Materials and Methods

### 2.1. Study Data

Publicly posted funding recommendations and appraisal documents for all oncology drug indications issued by Canadian Agency for Drugs and Technologies in Health (CADTH) in 2019 and 2020 were identified. Second, the websites of the National Institute for Health and Care Excellence (NICE) in the UK and Pharmaceutical Benefits Advisory Committee (PBAC) in Australia were searched to identify publicly posted recommendations matching the same drug and indication as those identified from CADTH. Any documents published by NICE and PBAC before the end of 2021 were considered for inclusion. The final study sample comprised oncology drug submissions, for which all three HTA agencies had issued a public reimbursement recommendation.

### 2.2. Data Abstraction

To facilitate comparison between the reporting and critical appraisal of the manufacturers’ economic models by the three HTA agencies, a set of commonly required attributes for economic evaluations submitted by drug manufacturers for reimbursement purposes was compiled from CADTH’s Guidelines for the Economic Evaluation of Health Technologies [4], the NICE Guide to the Methods of Technology Appraisals [5], and the Guidelines for Preparing a Submission to the PBAC [6]. Due to the focus of our study on oncology products, recommendations from NICE Decision Support Unit technical support document 14 (NICE DSU 14) [8] for the conduct and reporting of survival analysis for economic evaluation were also reviewed, as the NICE DSU 14 is explicitly referenced in CADTH and PBAC guidelines for economic evaluations of oncology indications. Recommendations from these appraisal guidelines were cross-referenced with the Consolidated Health Economic Evaluation Reporting Standards (CHEERS) statement [9] and economic evaluation guidelines from the International Society for Pharmacoeconomics and Outcomes Research (ISPOR) [10,11], in order to determine a minimum set of common reporting measures expected to be included in economic models submitted by manufacturers to CADTH, NICE, and PBAC. Based on this review, 21 common data elements expected to be described in manufacturer submissions to CADTH, NICE, and PBAC were identified, as shown in Table S2 in the Supplementary Online Material. Based on these common elements expected to be included in the economic models and reports submitted for reimbursement by manufacturers, an abstraction sheet was developed to capture to what extent CADTH, NICE, and PBAC report on characteristics of the manufacturer economic submissions, in terms of the type of analysis (e.g., cost-utility), utility value method for cost-utility analyses, model structure, time horizon, indirect comparison, equity issues, treatment of uncertainty, and validation of results. For interested readers, a glossary of technical terms is provided in Table S6 in the Supplementary Materials. For survival analyses and extrapolations, HTA reports were reviewed to document whether the following information was reported: whether a parametric approach was used, parametric distributions used for extrapolations, goodness-of-fit testing, testing of the proportional hazards assumption, curve fitting assessment, validation of extrapolations, treatment effect scenario analyses, justification for any use of external data, whether distributions were fitted to the tail of Kaplan–Meier curves or entirety of the curves, and whether or not alternative curve-fitting approaches were examined. A methodological element of interest was considered to be reported as long as it was mentioned in the HTA reports, irrespective of the quantity of information reported. If one HTA agency published multiple paragraphs describing a given category, the equity considerations, for example, while another HTA agency published a single sentence, they would both be categorized as having reported on equity. The data was abstracted by one reviewer, and 20% of the abstracted data was checked by a second reviewer.

HTA agencies’ methodological criticisms of the economic dossiers submitted by manufacturers were grouped into a set of seven categories: (1) time horizon; (2) treatment benefit; (3) utility values; (4) comparator; (5) subgroups; (6) progression-free survival estimates; (7) overall survival estimates; (8) costs; and (9) extrapolation of survival data. These common thematic categories were adapted from previous studies detailing nine methodological issues described in CADTH economic guidance reports [12] and ten common issues identified by CADTH’s economic guidance panel [13]. Incremental costs and QALYs reported in manufacturer submissions and re-calculated by the three HTA agencies were also documented. To facilitate appropriate comparison of incremental cost-effectiveness ratios (ICERs) between the three agencies, which use different currencies, ICERs reported by CADTH, NICE, and PBAC were converted to USD using 2021 purchasing power parity (PPP)-adjusted exchange rates published by the Organisation for Economic Co-operation and Development (OECD) [14]. Finally, the funding recommendation (list, do not list) was also abstracted for each oncology product evaluated by the three agencies in 2020 and 2021. Our initial expectation was that each of the elements described in each of the HTA agency submission guidelines would be summarized and reported in the published assessment reports, since they are required to be submitted by the manufacturer.

### 2.3. Data Analyses

In order to explore whether reporting of methodological approaches and sources of clinical evidence considered by each agency were similar, potential relationships between variables were assessed where appropriate. Dichotomous differences in the reporting of methods and recommendation outcomes were assessed using Chi-squared tests. Where appropriate, potential relationships between categorical variables, which include several modeling characteristics and survival curve extrapolation techniques, were substantiated through Chi-squared tests. Dichotomous differences in methodological criticisms observed between the three agencies were assessed using Cochran’s Q tests. For statistically significant Cochran’s Q test results, post hoc pairwise McNemar tests were conducted to identify pairwise relationships. To support the generalizability of our results, we conducted similar analyses for HTAs, which had recommendations available from only two of the three HTA agencies.

## 3. Results

### 3.1. Number of HTA Submissions Reviewed by CADTH between 2019–2020 Matched with Corresponding HTAs from NICE and PBAC

A total of 83 indications in oncology were identified from the CADTH website between 2019–2020. Matching these 83 indications with their corresponding public appraisal documents from NICE and the PBAC, 36 indications were found to have been reviewed by all three agencies, and these 36 indications (108 individual HTA appraisals) comprised our comparative study sample. Out of the 108 appraisals by NICE, CADTH, and PBAC, 14 recommendations were published by PBAC and NICE before the CADTH recommendations (i.e., before 2019), with 7 in 2021.

Of note, we excluded 17 indications (51 individual submission appraisals) that were evaluated by two of the three agencies, 19 indications (57 individual submission appraisals) that were evaluated by only one of the agencies, and 11 indications that were listed on the CADTH website, but for which none of the three agencies (including CADTH) had published a recommendation. Table S1 in the Supplementary Materials presents the list of indications/products reviewed by the three agencies, two agencies, and one agency only, as well as those indications for which no recommendations were issued.

### 3.2. Manufacturer Economic Submissions’ Characteristics

Table 1 presents the characteristics of the 108 economic evaluations, which were submitted to CADTH, NICE, and PBAC, as reported by these three HTA agencies in their public documents providing the rationale for the funding decision. Two thirds (67%) of the manufacturer economic submissions utilized a single phase 3 study as the main source of clinical data, and the most frequent therapeutic areas were lung cancer (25%) and leukemia (14%). Approximately two-thirds (64%) of the submissions were related to treatments for late-stage disease (stage IV or metastatic disease).

### 3.3. HTA Agency Reporting on Economic Model Characteristics Submitted by Manufacturers

As shown in Table 2, all three HTA agencies were consistent in their reporting of the basic characteristics of the economic models submitted by the manufacturers, in terms of the type of economic analyses, model structure, time horizon, treatment of uncertainty, and use of indirect treatment comparison used by the manufacturers, as these items were reported almost all the time by the three HTA agencies. However, some differences were observed between HTA agencies in the model characteristics submitted by the manufacturer or HTA reporting on some elements. Briefly, all submissions to CADTH and NICE were based on cost-utility techniques, while 17% of PBAC submissions were based on cost-minimization techniques (*p* = 0.013). Differences were observed between the HTA agencies, in terms of reporting the instrument used to derive the utility values for CADTH (44%), NICE (92%), and PBAC (61%) (*p* = 0.001), reporting on equity issues (*p* < 0.001) and which types of analyses were conducted to deal with uncertainty (*p* < 0.001), with NICE reporting this information more frequently than the CADTH and PBAC. Partitioned survival models were used in approximately 70% of the models, and most models used three health states. Indirect comparisons were used in more than half of the submissions.

### 3.4. HTA Agency Reporting on Methods Used to Extrapolate Survival Data in Manufacturers’ Cost-Effectiveness Models

Important numerical and statistical differences between the three HTA agencies were seen in the reporting of the methods used by manufacturers when analyzing and extrapolating survival data for cost-effectiveness modeling, with NICE reporting more often on the characteristics of the survival extrapolation methods used by manufacturers than CADTH and PBAC. For example, NICE consistently reported on whether parametric distributions were used (100% of the times), which statistical tests (e.g., AIC, BIC) were used to select the best fitting curves (94% of the times), whether the PH assumption was tested (89%), whether survival curves were fitted jointly or separately (86%), or if the extrapolations were validated (97%). In comparison, CADTH and PBAC discussed whether parametric distributions were used 56% and 78% of the time and rarely reported on the PH assumption (CADTH: 10% and PBAC: 32%). Table 3 presents the details, while Table 4 presents the parametric distributions used for modeling PFS and OS, as reported by the HTA agencies. Compared to NICE, who provided information on which statistical distributions were used, CADTH rarely reported which statistical distribution was used. While the Weibull, exponential, log-logistic, log-normal, and generalized gamma were used by manufacturers to model PFS or OS, no single distribution was being reported more than 25% of the time (Table 4).

### 3.5. HTA Agency Reporting on Methodological Criticisms of Manufacturer Economic Submissions

**Figure 1:**
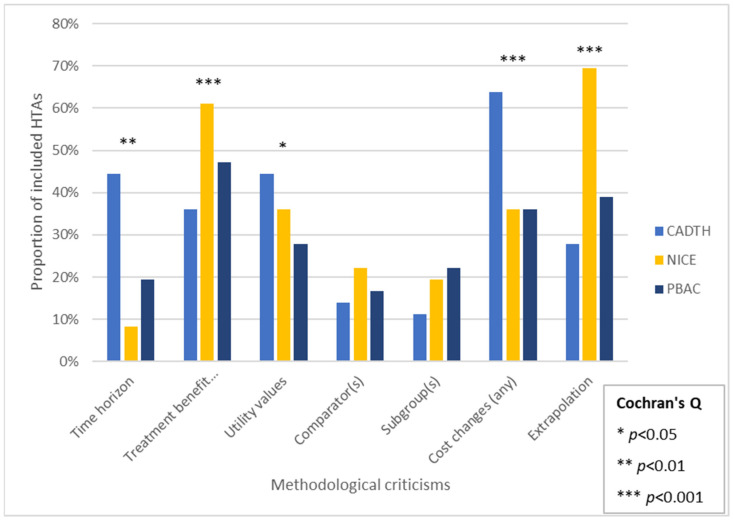
Methodological criticisms. Abbreviations: CADTH, Canada Agency for Drugs and Technologies in Health; PBAC, Pharmaceutical Benefits Advisory Committee; OS, overall survival; PFS, progression-free survival; NICE. National Institute for Health and Care Excellence.

In general, the three HTA agencies tended to focus on broadly similar areas of criticism, regarding the cost-effectiveness models for a given drug/indication, most often relating to the extrapolation of treatment benefit beyond the trial duration (CADTH: 36%, NICE: 47%, and PBAC: 39%), estimation of PFS (CADTH: 36%, NICE: 61%, and PBAC: 47%), and estimation of OS (CADTH: 53%, NICE: 61%, and PBAC: 44%). Notable differences between HTA agencies include NICE rarely criticizing manufacturers’ submitted time horizon (8%), while almost always criticizing extrapolations (69%), and CADTH usually criticizing both the manufacturers’ submitted time horizon (44%) and cost assumptions (64%) (Figure 1).

### 3.6. HTA Agency Reporting on Economic Results, HTA Economic Re-Analyses and Funding Recommendations

Table 5 presents the incremental QALY and incremental cost per QALY gained submitted by the manufacturers and following the re-analyses conducted by the HTA agencies. While all three HTA agencies reported the economic results submitted by the manufacturers, PBAC (50% of the time) and NICE (42%) redacted the QALYs results more often that CADTH (22%). Among those HTAs that reported unredacted QALYs, average incremental QALYs were of broadly similar magnitude across the three agencies, both for the manufacturer-submitted QALYs (CADTH: 1.30, NICE: 1.17, and PBAC: 1.52) and in the CADTH and NICE reanalyses of the model results (CADTH: 0.78 and NICE: 0.68) (Table 5). As the PBAC did not report the reanalysis of QALYs, no values were available for comparison. The average difference between the manufacturer-submitted and agency reanalyzed QALYs were also similar across agencies (CADTH: −60.3% and NICE: −58.5%). In terms of incremental cost-effectiveness ratios (ICERs), the ICERs expressed in the PPP were found to vary across the individual agencies, both in the manufacturer’s submitted estimates (CADTH: USD$110K/QALY, NICE: USD$66K/QALY, and PBAC: USD$49K/QALY) and agency reanalyses (CADTH: USD$201K/QALY and NICE: USD$113K/QALY). NICE and CADTH re-analyses almost doubled the ICER submitted by the manufacturer. In terms of recommendations, 94% of NICE recommendations were positive, 78% were positive for CADTH, and PBAC issued positive recommendations for 53% of the submissions. Statistical differences in recommendation status were seen between PBAC and NICE (*p* < 0.001) and PBAC and CADTH (*p* = 0.029). The three agencies issued the same recommendation (either positive or negative) in 39% of the included HTAs.

As a partial validation of the representativeness of our results, our supplementary analyses of economic appraisals, conducted by 2 of the 3 agencies (19 indications and 57 individual HTAs), confirmed the results of the main comparative study sample, as the observed frequencies of reporting among this alternative dataset (Tables S3–S5 in the Supplementary Materials) were broadly similar to those included in the main study.

## 4. Discussion

We hypothesized that consistency would be observed for the oncology medications evaluated by the three HTA agencies, due to the same product being assessed for the same indication, based on the same or similar clinical data.

### 4.1. Summary of Findings

We undertook a review of 36 oncology-based economic evaluations submitted by drug manufacturers for reimbursement purposes in Canada over the 2-year period, 2019–2020, which matched with corresponding submissions to the UK and Australia, for which an appraisal and funding recommendation report was publicly available from each HTA agency. Although we hypothesized that consistency of reporting would be observed due to the same product being assessed for the same indication, based on the same or similar clinical data, we found important differences in reporting. While the three HTA agencies consistently reported the baseline characteristics of these economic evaluations, NICE provided more information than CADTH or PBAC when describing the methods used for the extrapolations of survival data, despite the similar requirements for drug manufacturers to follow the same DSU guidelines [8]. Differences were also observed in the HTA agency criticisms of manufacturers’ submitted models and extent of the reanalysis undertaken. The level of detail provided by each HTA agency, as a rationale for their appraisal and funding recommendations, was also found to vary substantially. In general, NICE provided extensive documents that comprehensively detailed the clinical, economic, and technical aspects of manufacturer submissions, as well as in-depth assessment notes from the evidence review group (ERG). The appraisals by PBAC and CADTH, while providing relatively extensive review documents, nevertheless, did not provide the same level of detail and transparency as NICE. Both NICE and the PBAC, in contrast to CADTH, often redacted key outcomes in their HTAs (e.g., QALYs and ICERs). This discrepancy seems notable, given that all three agencies are publicly funded and assess the same drug products and indications using the same or very similar economic model. While each agency may approach their respective HTA process with a similar degree of rigor, it seems that the agencies have pursued different approaches in the quantity of reporting that they make available to the public.

This situation could be explained by different levels of resources assigned to the review of the economic evidence submitted by the manufacturers. For example, CADTH assigns a panel of external reviewers to critically appraise the information submitted by the manufacturer, while NICE utilizes a number of academic centers of excellence, the individual members of which may differ for each reimbursement submission. The PBAC is comprised of an independent statutory body of clinical and economic experts appointed by the Australian government. While criticisms of model assumptions varied across HTA agencies, the re-analyses conducted by CADTH and NICE to address model limitations nearly doubled the ICERs on average. It was difficult to assess ICERs re-analyzed by the PBAC, as these ICERs were presented as ranges with no point estimate, and often the range was quite wide for both manufacturer-submitted and PBAC re-analyzed ICERs. However, the percentage of positive recommendations were lower for PBAC than CADTH and NICE, which might be, at least partially, explained through different approaches to reimbursement (drug reimbursement in Australia does not include price negotiation, and the PBAC is instead a yes/no decision-making body).

### 4.2. Previous Studies

It is difficult to compare our study with the previous literature for several reasons. First, previous studies have examined jurisdictional differences across the published HTAs, focusing on the factors that influence HTA reimbursement recommendations from HTA agencies in Australia, Canada, England, and Scotland [1], differences in rates of positive and negative recommendations between Canada and the UK [15], and the impact of differing clinical evidence bases on the HTA recommendations from Australia, Canada, and the UK [16]. Each of these previous studies has been limited in scope, focusing predominantly on recommendation status across jurisdictions utilizing data that are now considerably dated (2014 or older [1]). Other studies have sought to identify relationships between HTA recommendations across jurisdictions, though most have been published more than 5 years ago [15,17,18,19,20,21,22,23], and are focused exclusively on one specific component of HTA submissions (e.g., surrogate endpoints) or one single disease area (e.g., schizophrenia) [18,24,25]. While a large majority of the previous studies have been focused on areas outside of oncology, we previously examined the published oncology literature regarding economic evaluation methods [7]. We showed that greater detail in reporting of survival analysis methods, including extrapolation, statistical analyses, and validation of results, is needed, in order to support greater consistency in decision making. To the authors’ knowledge, at the time of writing, no previous studies [1,17,19,21,26,27,28] have specifically examined how HTA agencies evaluate and report on economic evaluations submitted by manufacturers for reimbursement.

Our current study offers insights into the reporting by three HTA agencies across a broad spectrum of economic evaluation methods, including study characteristics, common economic evaluation attributes, survival analysis, recommendation status, and methodological criticisms. The differences in recommendation status we observed across 36 oncology indications, assessed by CADTH between 2019–2020, matched with corresponding HTAs from NICE and the PBAC, might be, at least partially, explained through different approaches to reimbursement. Nonetheless, our study does corroborate recent work [12,13], which showed that the time horizon and cost estimates were the most frequently criticized elements of manufacturer submissions to CADTH in the periods 2011–2014 and 2012–2018, respectively. However, our results also suggest that these criticisms may be unique to CADTH, as both NICE and PBAC were found to rarely criticize manufacturer-submitted time horizons (NICE: 8%, PBAC: 19%) or cost estimates (NICE: 36%, PBAC: 36%).

### 4.3. Limitations

This analysis provides useful insights into method reporting in HTA appraisal documents, but there are a number of important limitations that should be recognized. First, we conducted our study over a limited time period of 2 years (2019–2020); thus, publication bias may affect our results and conclusions. A different level of detail may have been reported in HTAs before 2019, and recent guideline updates or changes in the HTA review process at CADTH, NICE, and PBAC may impact what and how the HTA results are reported. For example, NICE announced, in 2021, an overhaul of methods to optimize evidence generation and global HTA strategy [29], while the Australian government has recently announced a new strategic agreement and the first independent review of Australia’s HTA system [30]. In addition, the Canadian study data included in our analyses was produced through the pCODR assessment pathway, which was specifically designed for review of cancer medications. In late 2020, CADTH announced a new review pathway, in which all submitted drugs, oncology or otherwise, would be reviewed under a single CADTH review procedure that would commence in 2021 [31]. Second, as we focused exclusively on oncology HTAs, caution should be exercised in generalizing our results to other therapeutic areas. Third, as long as an element of interest was mentioned in the HTA reports from CADTH, NICE, and PBAC, irrespective of the quantity of information reported, we considered it as reported. While not a specific objective of our study, the differences we observed in the quantity of information reported from agencies highlights the need for greater consistency in reporting for HTA bodies. We also converted the ICERs reported in the published HTAs across the three agencies, using purchasing power parity (PPP); however, it is difficult to directly compare the ICERs between regions, due to the differences in treatment costs or other relative prices. In addition, the PBAC reports only ranges of ICERs, rather than point estimates, which further inhibits the ability to compare PBAC ICERs with those from other HTA agencies. Finally, we assumed that the reimbursement submissions sent by manufacturers to CADTH, NICE, and the PBAC were similar, which may or may not be true. However, as demonstrated in our results, the main characteristics of the submissions to these three HTA agencies were observed to be similar.

It should be noted that our sample set included 36 indications in total (108 individual HTAs) that were available from all three HTA agencies out of an overall set of 83 indications (249 individual HTAs). In order to ensure that our comparative sample was representative, we analyzed public recommendations made by two agencies (e.g., N = 19 indications), and the results were consistent with the main analysis (N = 36 indications). From our research, it does appear that CADTH received a slightly higher number of HTA submissions than either NICE or PBAC. One speculative explanation could be that NICE and PBAC are known to be more restrictive in their assessments of submitted dossiers, and this may or may not have prompted a number of pharmaceutical manufacturers to not submit a reimbursement dossier to NICE and/or PBAC for some indications, due to a comparatively lower probability of success. Unfortunately, we cannot demonstrate or substantiate this point using our current research and dataset.

### 4.4. Future Directions

This study focuses on the recent recommendations published by three HTA agencies over a 2-year period and is, therefore, limited in both time horizon and scope. While 2 years was judged to be adequate for assessing the reporting of methods, and previous studies have used similar time scales and/or smaller sample sizes [12,13,32], future studies could be expanded to encompass HTA recommendations from additional years, in order to account for recent changes. Efforts could also be put into expanding comparisons beyond CADTH, NICE, and PBAC, in order to include other countries that have adopted HTA processes, such as South Korea, Taiwan, and, more recently, Japan. Our study data from 2019–2020 may provide a useful dataset for future comparisons with oncology drug submissions assessed under CADTH’s new procedures.

## 5. Conclusions

Based on our 2-year sample of oncology HTAs published by CADTH, NICE, and PBAC, the variations in the reporting we observed, especially for technical aspects, such as survival analysis, suggest that, in addition to the guidelines for HTA submissions, the community of HTA agencies should also have common standards for reporting the results of their assessments, though the information and opinions reported may differ.

## Figures and Tables

**Table 1:** Characteristics of included studies.

Characteristic	n	%
**HTA agency (n = 108)**		
pCODR	36	33%
NICE	36	33%
PBAC	36	33%
**Data source type (n = 108)**		
Ph3	79	67%
Ph2 (single arm)	16	15%
Mix of Ph3 and Ph2	4	3%
RWE	0	0%
Mix of Ph2 and RWE	5	6%
Mix of Ph3 and RWE	4	7%
Ph4	0	1%
**Type of cancer studied (n = 108)**		
Leukemia	15	14%
Breast	12	11%
Lung	27	25%
Genitourinary	9	8%
Gastrointestinal	12	11%
Lymphoma	6	6%
Skin and melanoma	12	11%
Other	3	3%
Myeloma	3	3%
Gynecology	6	6%
Head and neck	3	3%
Neurological	0	0%
**Cancer stage (n = 108)**		
Early/stage I	12	11%
Stage II/III	27	25%
Stage IV/metastatic	69	64%

Abbreviations: CADTH, Canada Agency for Drugs and Technologies in Health; HTA, health technology assessment; NICE, National Institute for Health and Care Excellence; PBAC, Pharmaceutical Benefits Advisory Committee; RWE, real-world evidence.

**Table 2:** Common economic evaluation attributes reported by HTA agencies (N = 108).

Reported Characteristic	Number of Studies n (%)			*p*-Value (χ2)
	CADTH	NICE	PBAC	
**Type of analysis**				
CUA	36 (100%)	36 (100%)	30 (83%)	0.013
CEA	0 (0%)	0 (0%)	0 (0%)	
Other (e.g., CMA)	0 (0%)	0 (0%)	6 (17%)	
**QALYs reported (Y/N)**				
Yes	34 (94%)	36 (100%)	30 (83%)	0.023
No	2 (6%)	0 (0%)	6 (17%)	
**Utility value method**				
EQ5D	15 (42%)	33 (92%)	18 (50%)	0.001
SF36	0 (0%)	0 (0%)	0 (0%)	
HUI	0 (0%)	0 (0%)	0 (0%)	
Other	1 (3%)	1 (3%)	4 (11%)	
Not reported	20 (56%)	2 (6%)	14 (39%)	
**Model structure**				
Partitioned survival	25 (69%)	25 (69%)	24 (67%)	0.112
Markov	11 (31%)	10 (28%)	6 (17%)	
Not reported	0 (0%)	0 (0%)	6 (17%)	
Decision tree	0 (0%)	0 (0%)	0 (0%)	
Combination (decision tree + Markov)	0 (0%)	1 (3%)	0 (0%)	
Other	0 (0%)	0 (0%)	0 (0%)	
**Number of modeled health states**				
Three	24 (67%)	29 (81%)	21 (58%)	0.516
Four	2 (6%)	3 (8%)	4 (11%)	
Five	4 (11%)	2 (6%)	1 (3%)	
Six	1 (3%)	0 (0%)	3 (8%)	
Seven or more	0 (0%)	2 (6%)	0 (0%)	
Not reported	5 (14%)	0 (0%)	7 (19%)	
**Time horizon (submitted by manufacturer)**				
1–5 years	4 (11%)	0 (0%)	3 (8%)	<0.001
6–10 years	14 (39%)	4 (11%)	16 (44%)	
11–20 years	7 (19%)	10 (28%)	3 (8%)	
21–30 years	3 (8%)	7 (19%)	3 (8%)	
31–40 years	2 (6%)	6 (17%)	3 (8%)	
40+ years	6 (17%)	8 (22%)	1 (3%)	
Not reported	0 (0%)	1 (3%)	7 (19%)	
**Indirect treatment comparison (Y/N)**				
Yes	20 (56%)	24 (67%)	20 (56%)	0.541
No	16 (44%)	12 (33%)	16 (44%)	
**Equity issues reported**				
Yes	0 (0%)	15 (42%)	0 (0%)	<0.001
No	36 (100%)	21 (58%)	36 (100%)	
**Handling of uncertainty**				
Deterministic sensitivity analysis	12 (33%)	33 (92%)	9 (25%)	<0.001
Probabilistic sensitivity analysis	11 (31%)	36 (100%)	4 (11%)	<0.001
Scenario analysis	13 (36%)	36 (100%)	27 (75%)	<0.001
**Validation (Y/N)**				
Yes	2 (6%)	35 (97%)	0 (0%)	<0.001
No	34 (94%)	1 (3%)	36 (100%)	
**Reimbursement recommendation**				
Reimburse	28 (78%)	34 (94%)	19 (53%)	<0.001

Abbreviations: CADTH, Canada Agency for Drugs and Technologies in Health; CEA, cost-effectiveness analysis; CMA, cost-minimization analysis; CUA, cost-utility analysis; EQ5D, European Quality of Life 5 dimensions; HUI, health utilities index; NICE, National Institute for Health and Care Excellence; PBAC, Pharmaceutical Benefits Advisory Committee; QALYs, quality-adjusted life-years; SF36, Short Form 36.

**Table 3:** Survival analysis attributes reported by HTA agencies.

Reported Characteristic	Number of Studies n (%)			*p*-Value (χ2)
	CADTH	NICE	PBAC	
**Parametric approach**				
Yes	20 (56%)	36 (100%)	28 (78%)	<0.001
No	16 (44%)	0 (0%)	8 (22%)	
**Standard parametric distributions tested**	**N = 20**	**N = 36**	**N = 28**	
Yes	17 (85%)	36 (100%)	21 (75%)	0.008
No	3 (15%)	0 (0%)	7 (25%)	
**Curve fitting assessment**	**N = 20**	**N = 36**	**N = 28**	
AIC	1 (5%)	2 (6%)	1 (4%)	<0.001
BIC	1 (5%)	0 (0%)	0 (0%)	
Both AIC and BIC	6 (30%)	30 (83%)	8 (29%)	
Other	0 (0%)	2 (6%)	1 (4%)	
Not reported	28 (60%)	2 (6%)	26 (64%)	
**PH assumption tested (if appropriate)**	**N = 20**	**N = 36**	**N = 28**	
Yes	2 (10%)	32 (89%)	9 (32%)	<0.001
No	18 (90%)	4 (11%)	19 (68%)	
**Fitted parametric curves**	**N = 20**	**N = 36**	**N = 28**	
Jointly fitted models	1 (5%)	20 (56%)	10 (36%)	<0.001
Separately fitted models	0 (0%)	11 (31%)	4 (14%)	
Not reported	19 (95%)	5 (14%)	14 (50%)	
**Validation of extrapolations**				
Yes	1 (3%)	35 (97%)	6 (17%)	<0.001
No	35 (97%)	1 (3%)	30 (83%)	
**Scenario analyses of treatment effect**				
Yes	12 (33%)	19 (53%)	11 (31%)	0.109
No	24 (67%)	17 (47%)	25 (69%)	
**Use/source of external data justified**				
Yes	1 (3%)	20 (56%)	4 (11%)	<0.001
No	35 (97%)	16 (44%)	32 (89%)	
**Curves fitted to tail of Kaplan–Meier curves only**				
Yes	1 (3%)	4 (11%)	3 (8%)	0.389
No	35 (97%)	32 (89%)	33 (92%)	
**Alternative curve-fitting approaches examined**				
Yes	3 (8%)	9 (25%)	1 (3%)	0.011
No	33 (92%)	27 (75%)	35 (97%)	

Abbreviations: CADTH, Canada Agency for Drugs and Technologies in Health; HTA, health technology assessment; NICE, National Institute for Health and Care Excellence; OS, overall survival; PBAC, Pharmaceutical Benefits Advisory. Committee; PFS, progression-free survival.

**Table 4:** Parametric distributions selected for survival curve extrapolations.

Selected Parametric Curve Reported	Treatment						Comparator					
	CADTH (n = 20)		NICE (n = 36)		PBAC (n = 28)		CADTH (n = 20)		NICE (n = 36)		PBAC (n = 28)	
	PFS	OS	PFS	OS	PFS	OS	PFS	OS	PFS	OS	PFS	OS
Weibull	5%	10%	22%	17%	11%	14%	0%	5%	22%	14%	11%	14%
Exponential	0%	5%	8%	25%	25%	32%	0%	5%	8%	25%	25%	25%
Log-logistic	0%	5%	17%	19%	7%	11%	0%	0%	17%	19%	11%	14%
Log-normal	15%	5%	19%	17%	32%	14%	10%	5%	17%	17%	25%	18%
Gamma	0%	0%	0%	0%	0%	0%	0%	0%	3%	0%	0%	0%
Generalized gamma	0%	0%	14%	6%	11%	4%	0%	5%	11%	3%	11%	4%
Gompertz	5%	0%	8%	6%	0%	7%	0%	0%	8%	6%	0%	7%
Other	0%	0%	0%	3%	0%	0%	0%	0%	0%	3%	0%	0%
Not reported	75%	75%	11%	8%	14%	18%	90%	80%	14%	14%	18%	18%

Abbreviations: CADTH, Canada Agency for Drugs and Technologies in Health; HTA, health technology assessment; NICE, National Institute for Health and Care Excellence; OS, overall survival; PBAC, Pharmaceutical Benefits Advisory. Committee; PFS, progression-free survival.

**Table 5:** Comparison of manufacturer and agency-reanalyzed incremental quality-adjusted life-years and incremental cost-effectiveness ratios (ICERs).

HTA Agency	Incremental QALYs					
	Manufacturer: Base Case	Range		Agency Re-Analysis: Base Case	Range	Average Change
CADTH (n = 32)	1.30	0.13 to 4.34	CADTH (n = 28)	0.78	0.08 to 2.25	−60.3%
NICE (n = 21)	1.17	0.07 to 3.44	NICE (n = 15)	0.68	0.07 to 2.75	−58.5%
PBAC (n = 18)	1.52	0.13 to 6.84	N/A	N/A	N/A	N/A
**HTA Agency**	**ICER**					
	**Manufacturer:** **Base Case**	**Range**		**Agency Re-Analysis**	**Range**	**Average Change**
CADTH (n = 32)	$109,581	$12,242 to $388,172	CADTH (n = 32)	$200,923	$41,414 to $983,977	183.4%
NICE (n = 27)	$65,778	$6631 to $137,200	NICE (n = 26)	$112,891	$23,744 to $229,381	171.6%
PBAC (n = 23)	$48,665	$18,910 to $129,217	N/A	N/A	N/A	N/A

Abbreviations: CADTH, Canada Agency for Drugs and Technologies in Health; HTA, health technology assessment; ICER, incremental cost-effectiveness ratio; NICE, National Institute for Health and Care Excellence; PBAC, Pharmaceutical Benefits Advisory Committee; PPP, purchasing power parity; QALY, quality-adjusted life-year.

## Data Availability

The data presented in this study are available upon reasonable request from the corresponding author.

## Supplementary Materials

The following supporting information can be downloaded at: https://www.mdpi.com/article/10.3390/curroncol29100602/s1: Table S1. A total of 83 publicly available therapeutic indications and availability of corresponding HTA documents from CADTH, NICE, and the PBAC. Table S2: Common economic evaluation attributes from existing guidelines. Table S3. Selected results from alternative dataset: main source of clinical data. Table S4. Selected results from alternative dataset: economic evaluation attributes. Table S5. Selected results from alternative dataset: survival analysis. Table S6. Glossary of technical terms.
