# Supplementary material for: Appraisals by Health Technology Assessment Agencies of Economic Evaluations Submitted as Part of Reimbursement Dossiers for Oncology Treatments: Evidence from Canada, the UK, and Australia
Source: Curr Oncol. 2022 Oct 13;29(10):7624–36. doi: 10.3390/curroncol29100602 (PMC9600934; doi:10.3390/curroncol29100602)
Supplement: Supplementary file 1 [file curroncol-29-00602-s001.zip › curroncol-1939681-supplementary.pdf]

Supplementary Materials

Table S1. 83 publicly available therapeutic indications and availability of corresponding HTA documents from CADTH, NICE, and the PBAC

Drug (trade name)	Drug (generic name)	Product / indication assessed by:				Disease area
		0 agencies (n=11)	1 agency (n=19)	2 agencies (n=17)	3 agencies (n=36)	
Adcetris	brentuximab vedotin				***	Peripheral t-cell lymphoma
Kisqali	ribociclib		*			Advanced or metastatic breast cancer
Tecentriq & Avastin	atezolizumab & bevacizumab				***	Hepatocellular Carcinoma
Zejula	niraparib				***	Ovarian Cancer
Keytruda	pembrolizumab				***	Head and Neck Squamous cell Carcinoma
Tecentriq & Avastin	atezolizumab & bevacizumab				***	Non-Squamous Non-Small Cell Lung Cancer
Xospata	gilteritinib			**		Acute Myeloid Leukemia
Calquence	acalabrutinib			**		Chronic Lymphocytic Leukemia
Odomzo	sonidegib			**		Basal Cell Carcinoma
Venclexta	venetoclax				***	Chronic Lymphocytic Leukemia
Cabometyx	cabozantinib		*			Hepatocellular Carcinoma
Erleada	apalutamide		*			Metastatic Castration-Sensitive Prostate Cancer

Kisqali	ribociclib with fulvestrant				***	advanced or metastatic breast cancer
Nubeqa	darolutamide				***	Non-Metastatic Castration Resistant Prostate Cancer
Daurismo	glasdegib		*			Acute Myeloid Leukemia
Blincyto	blinatumomab				***	Minimal Residual Disease-Positive B-Cell Precursor Acute Lymphoblastic Leukemia
Calquence	acalabrutinib				***	Chronic Lymphocytic Leukemia (previously untreated)
Xtandi	enzalutamide			**		Metastatic Castration-Sensitive Prostate Cancer
Adcetris	brentuximab vedotin		*			Stage IV Hodgkin lymphoma
Keytruda	pembrolizumab			**		Renal Cell Carcinoma
Mylotarg	gemtuzumab ozogamicin				***	Acute Myeloid Leukemia
Rydapt	midostaurin		*			Systemic Mastocytosis
Adcetris	brentuximab vedotin				***	Primary Cutaneous Anaplastic Large Cell Lymphoma or CD30-Expressing Mycosis Fungoides
Rozlytrek	entrectinib				***	ROS1-positive Non-Small Cell Lung Cancer
Lonsurf	trifluridine-tipiracil				***	Gastric Cancer
Darzalex	daratumumab		*			Myeloma
Lynparza	olaparib					BRCA-mutated HER2-negative metastatic breast cancer

Keytruda	pembrolizumab					Metastatic microsatellite instability high or mismatch repair deficient endometrial cancer
Keytruda	pembrolizumab					Metastatic microsatellite instability high or mismatch repair deficient colorectal cancer
Lorbrena	lorlatinib				***	Non-Small Cell Lung Cancer
Inrebic	fedratinib		*			Myelofibrosis
Tecentriq	atezolizumab				***	Small Cell Lung Cancer
TBD	entrectinib		*			Neurotrophic Tyrosine Receptor Kinase Fusion-Positive Solid Tumours
Tecentriq	atezolizumab			**		Advanced or Metastatic Triple-Negative Breast Cancer
Kadcyla	trastuzumab emtansine				***	Early Breast Cancer
Libtayo	cemiplimab				***	Cutaneous Squamous Cell Carcinoma
Keytruda	pembrolizumab			**		Squamous Non-Small Cell Lung Cancer
Lynparza	olaparib				***	Ovarian Cancer
Nerlynx	neratinib				***	Hormone Receptor-Positive Breast Cancer
Lonsurf	trifluridine-tipiracil				***	Metastatic Colorectal Cancer
Idhifa	enasidenib		*			Acute Myeloid Leukemia
Vitrakvi	larotrectinib				***	Neurotrophic Tyrosine Receptor Kinase Locally Advanced or Metastatic Solid Tumours
Zytiga	abiraterone					Prostate Cancer
Atriance	nelarabine					Acute Lymphoblastic Leukemia

Keytruda	pembrolizumab		*			Metastatic Urothelial Carcinoma
Pomalyst	pomalidomide			**		Multiple Myelom
Darzalex	daratumumab		*			Multiple Myeloma
Alunbrig	brigatinib				***	Non-Small Cell Lung Cance
Keytruda	pembrolizumab				***	Melanoma Adjuvant Treatment
Lutathera	lutetium Lu 177 dotatate			**		Gastroenteropancreatic neuroendocrine tumors
Lenvima	lenvatinib				***	Hepatocellular Carcinoma
Bosulif	bosutinib		*			Chronic Myeloid Leukemia
Ninlaro	ixazomib			**		Multiple Myeloma
Verzenio	abemaciclib				***	Advanced or metastatic breast cancer
Imbruvica	ibrutinib		*			Chronic Lymphocytic Leukemia/Small Lymphocytic Leukemia
Imbruvica	ibrutinib					Waldenstrom's Macroglobulinemia
Revlimid	lenalidomide				***	Multiple Myeloma
Keytruda	pembrolizumab				***	Non-Squamous Non-Small Cell Lung Cancer
Oncaspar	pegaspargase					Adult Acute Lymphocytic Leukemia
Zirabev	bevacizumab (biosimilar)		*			Metastatic Colorectal Cancer; Non-Small Cell Lung Cancer
Venclexta	venetoclax				***	Chronic Lymphocytic Leukemia
Vizimpro	dacomitinib			**		Non-Small Cell Lung Cancer
Zevalin	ibritumomab					Non-Hodgkin's Lymphoma

Trazimera	trastuzumab (biosimilar)		*			Breast and Gastric Cancer Biosimilar
Truxima	rituximab (biosimilar)		*			Non-Hodgkin’s Lymphoma and Chronic Lymphocytic Leukemia
Xalkori	crizotinib				***	ROS1-positive Non-Small Cell Lung Cancer
Ogivri	trastuzumab (biosimilar)		*			Early Breast Cancer / Metastatic Breast Cancer / Metastatic Gastric Cancer
Demylocan	decitabine					Myelodysplastic Syndromes
Ibrance	palbociclib			**		Advanced or Metastatic Breast Cancer
Imfinzi	durvalumab				***	Non-Small Cell Lung Cancer
Tafinlar & Mekinist	dabrafenib & trametinib				***	Melanoma Adjuvant Treatment
Zytiga	abiraterone					Prostate Cancer
Not reported	rituximab (biosimilar)					Non-Hodgkin's Lymphoma and Chronic Lymphocytic Leukemia
Lenvima	lenvatinib				***	Renal Cell Carcinoma
Blincyto	blinatumomab			**		Philadelphia chromosome positive B-cell precursor acute lymphoblastic leukemia
Folotyn	pralatrexate			**		Peripheral t-cell lymphoma
Unituxin	dinutuximab			**		Neuroblastoma
Xtandi	enzalutamide			**		Non-metastatic castration-resistant prostate cancer
Adcetris	brentuximab Vedotin			**		Hodgkin Lymphoma

Opdivo	nivolumab				***	Melanoma Adjuvant Therapy
Cabometyx	cabozantinib				***	Renal Cell Carcinoma
Tagrisso	osimertinib				***	Non-Small Cell Lung Cancer
Mvasi	bevacizumab (biosimilar)		*			Metastatic Colorectal Cancer / Non-Small Cell Lung Cancer

Abbreviations: CADTH, Canada Agency for Drugs and Technologies in Health; NICE, National Institute for Health and Care Excellence; PBAC, Pharmaceutical Benefits Advisory Committee

Table S2: Common economic evaluation attributes from existing guidelines

Extraction element	Synthesis of common categories	CADTH[4] Guidelines	NICE[5] Guidelines	PBAC[6] Guidelines	AMCP[33] Guidelines	CHEERS[9] Checklist	ISPOR Guidelines[10]	ISPOR Guidelines[11]
Perspective	**Perspective**							
Indication	**Indication**							
Target population	**Target population**							
Subgroups	**Subgroup analysis**							
Comparator(s)	**Choice of comparator**							
Time horizon	**Time horizon**							
Type of analysis	**Preferred analytical technique**							
Types of costs	**Costs to be included**							

Model structure	**Modeling**							
SLR (Y/N)	**Systematic review of evidences**							
QALYs (Y/N)	**Preference for effectiveness over efficacy**							
	**Preferred outcome measure stated**							
Utility value method	**Preferred method for deriving utility values**							
Equity	**Equity issues stated**							
Discount rate	**Discounting costs**							
Discount rate	**Discounting outcomes**							
PSA, DSA, scenarios	**Sensitivity analysis-methods**							
Incremental (Y/N)	**Incremental analysis**							
ICERs/ICURs	**Total costs vs effectiveness**							
Validation (Y/N)	**Portability of results (Generalizability)**							
BIA (Y/N)	**Financial impact analysis**							

Survival analysis methods	**Survival analysis**							

	Included
	Not included in published academic guidelines

Abbreviations: CADTH, Canada Agency for Drugs and Technologies in Health; DSA, deterministic sensitivity analysis; ICER, incremental cost-effectiveness ratio; ICUR, incremental cost-utility ratio; N, No; N/A, Not applicable; NICE, National Institute for Health and Care Excellence; PBAC, Pharmaceutical Benefits Advisory Committee; PSA, probabilistic sensitivity analysis; QALY, quality-adjusted life-year; SLR, systematic literature review; Y, Yes.

Table S3. Selected results from alternative dataset: main source of clinical data

Characteristic	N	%	Number of studies n (%)
**Data source type**			
Phase 3 trial	29	55%	29 (55%)
Phase 2 trial (single arm)	8	15%	8 (15%)
Mix (Phase 2 and 3 trials)	1	4%	1 (4%)
RWE	0	15%	0 (15%)
Mix (Phase 2 trial and RWE)	5	9%	5 (9%)
Mix (Phase 3 trial and RWE)	8	2%	8 (2%)
Phase 4	2	0%	2 (0%)

Abbreviations: RWE, real-world evidence

Table S4. Selected results from alternative dataset: economic evaluation attributes

Reported characteristic	Number of studies n (%)		
	CADTH	NICE	PBAC
**Type of analysis**			
CUA	19 (66%)	11 (100%)	8 (62%)
CEA	8 (28%)	0 (0%)	0 (0%)
Other (e.g. CMA)	2 (3%)	0 (0%)	5 (31%)
**Model structure**			
Partitioned survival	21 (75%)	9 (82%)	3 (23%)
Markov	4 (14%)	2 (18%)	1 (8%)
Decision tree	0 (0%)	0 (0%)	0 (0%)
Combination (decision tree + Markov)	2 (7%)	0 (0%)	1 (8%)
Other	1 (4%)	0 (0%)	0 (0%)
Not reported	1 (4%)	0 (0%)	8 (62%)
**Reimbursement recommendation**			
Reimburse	22 (76%)	9 (82%)	12 (0%)
Do not reimburse	7 (24%)	2 (18%)	1 (100%)

Abbreviations: CADTH, Canada Agency for Drugs and Technologies in Health; CEA, cost-effectiveness analysis; CMA, cost minimization analysis; CUA, cost-utility analysis; NICE, National Institute for Health and Care Excellence; PBAC, Pharmaceutical Benefits Advisory Committee

Table S5. Selected results from alternative dataset: survival analysis

Reported characteristic	Number of studies n (%)		
	CADTH (N=29)	NICE N=11	PBAC (N=13)
**Parametric approach**			
Yes	16 (55%)	11 (100%)	4 (31%)
No	13 (45%)	0 (0%)	9 (69%)
**Curve fitting assessment**	**N=16**	**N=11**	**N=4**
AIC	1 (3%)	0 (0%)	0 (0%)
BIC	1 (3%)	0 (0%)	0 (0%)
Both AIC and BIC	6 (21%)	10 (91%)	2 (50%)
Other	0 (0%)	1 (9%)	0 (0%)
Not reported	8 (72%)	0 (0%)	2 (50%)

Abbreviations: CADTH, Canada Agency for Drugs and Technologies in Health; NICE, National Institute for Health and Care Excellence; PBAC, Pharmaceutical Benefits Advisory Committee

Table S6. Glossary of technical terms

Term	Abbreviation	Description
cost-effectiveness acceptability curve	CEAC	A graphical representation of the uncertainty associated with the results of an economic evaluation. It plots for a range of cost effectiveness thresholds against the probability that the new technology /intervention will be cost effective at that threshold. This helps decision-makers understand the uncertainty surrounding the optimal treatment strategy.
cost-effectiveness analysis	CEA	A form of economic evaluation that is best suited to addressing questions of technical efficiency. Comparisons are limited to services or treatment options that produce the same type of benefit, which is valued strictly in one-dimensional, natural units.
cost-minimization analysis	CMA	A special type of cost-effectiveness analysis, which is possible only if it has been determined (or more often assumed) that there are no differences in benefits between the alternate interventions compared and thus the evaluation is based on only the costs of the interventions.
cost-utility analysis	CUA	A variant of cost-effectiveness analysis where the health outcome measure of interest is usually expressed as a quality adjusted life year, a single index that combines length of life and a quality adjustment for less than perfect health (i.e. the utility score).
health technology assessment	HTA	A multidisciplinary process that uses explicit methods to determine the value of a health technology at different points in its lifecycle. The purpose is to inform decision making in order to promote an equitable, efficient, and high-quality health system.

incremental cost-effectiveness ratio	ICER	The ratio of the difference in costs between an intervention and a specified comparator to the difference in effectiveness between that intervention and the specified comparator. From the results of a cost-effectiveness analysis, an incremental cost-effectiveness ratio can be calculated that depicts the extra cost per unit of outcome obtained, in comparing one treatment option to another.
network meta-analysis	NMA	A technique used in systematic reviews to compare the relative effectiveness of three or more interventions simultaneously that have not been compared in a single randomised trial or a single analysis by combining both direct and indirect effectiveness across a network of studies.
probabilistic sensitivity analysis	PSA	Probabilistic sensitivity analysis represents parameters (inputs) as distributions of possible mean values instead of single point estimates.
quality-adjusted life-year	QALY	A measure of health outcome, which captures both length of life and the quality of life. QALYs are calculated by multiplying the total time (years) in a specific health state (or the number of life years remaining) by the "utility" of those years (measured from zero, representing the worst imaginable health (values less than zero represents health states worse than death), to one, representing perfect health).
